# The Optimal Therapy after Progression on Immune Checkpoint Inhibitors in MSI Metastatic Gastrointestinal Cancer Patients: A Multicenter Retrospective Cohort Study

**DOI:** 10.3390/cancers14205158

**Published:** 2022-10-21

**Authors:** Mifen Chen, Zhenghang Wang, Zimin Liu, Ning Liu, Weijia Fang, Hangyu Zhang, Xuan Jin, Jiayi Li, Weifeng Zhao, Huajun Qu, Fanghua Song, Zhiwei Chang, Yi Li, Yong Tang, Chunlei Xu, Xiaotian Zhang, Xicheng Wang, Zhi Peng, Jinping Cai, Jian Li, Lin Shen

**Affiliations:** 1 Key Laboratory of Carcinogenesis and Translational Research (Ministry of Education), Department of Gastrointestinal Oncology, Peking University Cancer Hospital & Institute, 52 Fucheng Road, Haidian District, Beijing 100142, China; 2 Department of Oncology, The Affiliated Hospital of Qingdao University, Qingdao 266003, China; 3 Department of Medical Oncology, First Affiliated Hospital, School of Medicine, Zhejiang University, Hangzhou 310058, China; 4 Department of Medical Oncology, Peking University First Hospital, Beijing 100034, China; 5 Department of Medical Oncology, The First Affiliated Hospital of Xiamen University, School of Medicine, Xiamen University, Xiamen 361005, China; 6 Department of Oncology, Henan Provincial People’s Hospital/People’s Hospital of Zhengzhou University/People’s Hospital of Henan University, Zhengzhou 450001, China; 7 Department of Medical Oncology, The Affiliated Yantai Yuhuangding Hospital of Qingdao University, Yantai 264099, China; 8 Department of Oncology, Dalian University Affiliated Xinhua Hospital, Dalian 116021, China; 9 Department of Oncology, The First Affiliated Hospital of Zhengzhou University, Zhengzhou 450052, China; 10 Department of Digestive Internal Medicine, The Affiliated Tumor Hospital of Xinjiang Medical University, Urumqi 830011, China; 11 Medical Affairs, 3D Medicines, Inc., Shanghai 201321, China

**Keywords:** gastrointestinal cancers, MSI/dMMR, immune checkpoint inhibitors, resistance

## Abstract

**Simple Summary:**

Programmed death 1 (PD1)/programmed death ligand-1 (PD-L1) inhibitor is the standard therapy for advanced microsatellite instability (MSI)/mismatch repair-deficient (dMMR) gastrointestinal cancers. However, the suitable therapy after the progression of anti-PD1/PD-L1 for MSI/dMMR gastrointestinal cancer patients was unknown, until now. Here, we conducted a retrospective study to evaluate the efficacy of anti-PD1/PD-L1 plus other drug therapy versus chemotherapy with or without targeted therapy for patients who had progressed on prior anti-PD1/PD-L1 monotherapy. Our study found that anti-PD1/PD-L1 plus other drug therapy had significantly improved the disease control rate, progression-free survival, and overall survival, along with a numerically higher objective response rate versus chemotherapy with or without targeted therapy. The promising findings of our retrospective study need to be further confirmed in prospective trials.

**Abstract:**

Background: In microsatellite instability (MSI)/mismatch repair-deficient (dMMR) gastrointestinal cancers, the optimum therapy after the progression of immune checkpoint inhibitors (ICIs) is yet unknown. Here, we compared the efficacy of programmed death 1 (PD1)/programmed death ligand-1 (PD-L1) inhibitors plus other therapy and chemotherapy with or without targeted therapy in MSI/dMMR gastrointestinal cancer patients after progression on anti-PD1/PD-L1 monotherapy. Methods: We retrospectively recruited MSI/dMMR gastrointestinal cancer patients who had progressed on anti-PD1/PD-L1 monotherapy. Objective response rate (ORR), disease control rate (DCR), progression-free survival (PFS), overall survival (OS), and PFS ratio (PFSr) were compared between patients who received anti-PD1/PD-L1 plus other therapy (ICI-plus group) and patients who received chemotherapy with or without targeted therapy (chemo-targeted group). Results: In total, 26 and 25 patients were recruited in the ICI-plus group and chemo-targeted group, respectively. Significantly better DCR (80.8% vs. 44.0%, *p* = 0.007), PFS (median PFS 6.9 months vs. 3.0 months, *p* = 0.001), OS (median OS NR vs. 14.1 months, *p* = 0.043), and PFSr (2.4 vs. 0.9, *p* = 0.021), along with a numerically higher ORR (23.1% vs. 12.0%, *p* = 0.503) were observed in the ICI-plus group compared with the chemo-targeted group. Multivariate analyses identified the therapy regimen as an important prognostic factor in gastrointestinal cancers. Conclusions: Compared to conventional chemotherapy with or without targeted therapy, continuing anti-PD1/PD-L1 in combination with other treatments showed better clinical outcomes in MSI/dMMR gastrointestinal cancer patients who progressed on PD1/PD-L1 blockade, which should be validated prospectively in clinical trials.

## 1. Introduction

Gastrointestinal cancers are highly prevalent worldwide, especially colorectal cancer (CRC) and gastric cancer (GC). According to the Global Cancer Statistics 2020, the incidence rate of CRC and GC ranked third and fifth globally, respectively. It was reported that 9.9 million patients died of cancer in 2020, of which 9.4% and 7.7% died of CRC and GC, respectively [1]. The disease burden of gastrointestinal cancers is also heavy in China, with CRC, GC, and liver cancer accounting for three of the top four cancers in incidence and mortality [2].

Microsatellite instability (MSI), known as a status of mismatch repair protein-deficient (dMMR), appears in approximately 5–10% of advanced CRC and GC patients [3]. Due to the deficiency of the mismatch repair function, MSI/dMMR causes an accumulation of mutations that result in neoantigen formation [4]. As a result, patients with MSI/dMMR are sensitive to immune checkpoint inhibitors (ICIs) [5]. Nowadays, programmed cell-death protein 1 (PD1)/programmed cell-death 1 ligand 1 (PD-L1) inhibitors have been set as standard therapy in MSI/dMMR cancers [6]. Moreover, pembrolizumab, an anti-PD1 antibody, has been approved as the first-line therapy in MSI/dMMR CRC patients based on the impressive results of KEYNOTE177 [7]. PD1/PD-L1 inhibitors have greatly improved the prognosis of MSI/dMMR gastrointestinal patients, with an objective response rate (ORR) of 30–40%, and a durable response is observed in some patients [8,9]. However, over 50% of patients still experience primary or acquired resistance to anti-PD1/PD-L1 therapy.

Previous studies demonstrated that the mechanisms of immune resistance to ICIs might involve mutations in the Wnt/β-catenin pathway [10], PI3K-AKT-mTOR pathway [11,12], CDH1 gene [13], elevated expression of Dickkopf 1 (DKK1) [14], and heterogeneity in tumor mutation burden and tumor microenvironment [15]. Although the exact mechanisms of resistance to ICIs remain to be clarified, how to effectively treat MSI/dMMR gastrointestinal cancers after developing progression on ICIs is an urgent problem to be solved. Thus far, limited open literature has reported the therapy strategy in MSI/dMMR gastrointestinal patients who have received prior ICI monotherapy. There are only two studies prospectively exploring the efficacy of anti-PD1/PD-L1 plus inhibitors of some emerging targets, including TGF (15 patients) [16] and TIM3 (22 patients) [17], and a retrospective study analyzing the efficacy of conventional chemotherapy with or without targeted therapy (31 patients) [18]. Regrettably, they all failed. Recently, the latest data on KEYNOTE177 showed that 14 and 38 patients received subsequent therapy with and without anti-PD1/PD-L1 after progression on pembrolizumab, respectively, but the associated efficacy was still unknown [19].

Here we retrospectively collected data on therapy post-ICI from multiple centers to compare the efficacy of anti-PD1/PD-L1 plus other therapy versus conventional chemotherapy with or without targeted therapy for MSI/dMMR gastrointestinal cancer patients who had progressed on prior anti-PD-1/PD-L1 monotherapy. We hope our study can provide some significant references for clinical practice to help improve the prognosis of MSI/dMMR gastrointestinal cancer patients.

## 2. Methods

### 2.1. Patients

**Figure 1:**
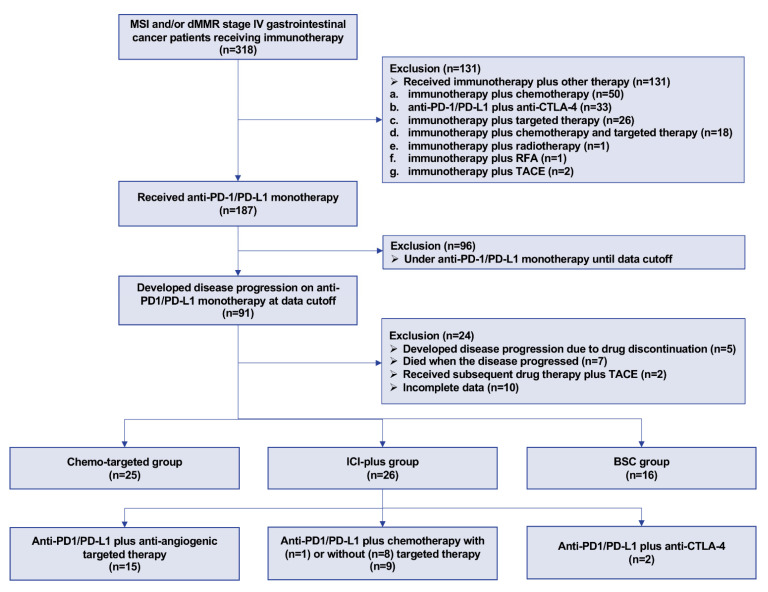
Flow chart of patient selection. Abbreviations: ICI-plus: anti-PD1/PD-L1 plus other therapy; chemo-targeted: chemotherapy with or without targeted therapy; BSC: best supportive care; CTLA-4 = cytotoxic T lymphocyte-associated antigen-4; dMMR = mismatch repair-deficient; MSI = microsatellite instability; PD1 = programmed death 1; PD-L1 = programmed death ligand-1; RFA = radiofrequency ablation; TACE = transcatheter arterial chemoembolization.

We retrospectively collected data from 10 centers across China between November 2016 and February 2022. Patients who met the following criteria were included: (1) pathologically confirmed gastrointestinal cancers; (2) diagnosed as stage IV; (3) confirmed as MSI detected by polymerase chain reactions (PCR) or next-generation sequencing (NGS), or confirmed as dMMR detected by immunohistochemicals (IHC). On the occasion of contradiction between results of PCR/NGS and IHC, the former shall prevail; (4) had received prior anti-PD1/PD-L1 monotherapy and developed disease progression based on the Response Evaluation Criteria In Solid Tumors (RECIST), version 1.1 [20]; (5) received subsequent drug therapy, including anti-PD1/PD-L1 plus other drug therapy, chemotherapy with or without targeted therapy, or best supportive care (BSC) after progression on prior anti-PD/PD-L1 monotherapy. For patients receiving subsequent anti-PD1/PD-L1 plus other drug therapy, anti-PD1/PD-L1 plus chemotherapy, targeted therapy, and anti-cytotoxic T lymphocyte antigen 4 (CTLA-4) are all allowed. The exclusion criteria included: (1) received initial anti-PD1/PD-L1 combined with other therapy, including but not limited to receiving anti-PD1/PD-L1 plus chemotherapy, targeted therapy, anti-CTLA-4, or local therapy, such as radiotherapy, radiofrequency ablation, and transcatheter arterial chemoembolization (TACE); (2) terminated prior anti-PD1/PD-L1 monotherapy due to adverse events or other reasons, and the investigators judged that the progression resulted from drug discontinuation; (3) died at the time of disease progression on anti-PD1/PD-L1 monotherapy; (4) after progression on prior anti-PD/PD-L1 monotherapy, received subsequent drug therapy plus local therapy, such as TACE; (5) lacked detailed therapy data after progression on anti-PD1/PD-L1. The flow chart of patient inclusion and exclusion is presented in Figure 1.

### 2.2. Outcome Evaluation

For patients who received post-ICI drug therapy, efficacy was evaluated regularly through computed tomography (CT) or magnetic resonance imaging (MRI) scan, and documented as complete response (CR), partial response (PR), stable disease (SD), or progressive disease (PD) according to RECIST 1.1. Outcomes evaluated in our study included ORR, disease control rate (DCR), progression-free survival (PFS), overall survival (OS), and PFS ratio (PFSr) in patients who received post-ICI drug therapy, and OS in patients who received BSC. ORR was defined as the proportion of patients who achieved the best response of CR or PR, and DCR was defined as the proportion of patients whose best response was CR, PR, or SD. PFS represented the time from the start of post-ICI therapy to disease progression or death, whichever happened first. For patients who received drug therapy, OS was measured as the time from the start of post-ICI therapy to death, and for patients who received BSC, OS was measured as the time from the day of progression on prior anti-PD1/PD-L1 monotherapy to death. PFSr was a novel index to reflect the interaction among diverse therapy lines and was defined as the PFS of the post-ICI therapy divided by the PFS of the prior anti-PD1/PD-L1 monotherapy, whereas a PFSr of more than 1.3 was considered as a treatment benefit [21,22]. Moreover, we defined primary resistance to prior anti-PD1/PD-L1 monotherapy as PD at the first scan or SD for less than 6 months, while acquired resistance was defined as PD after an initial response (PR or CR) or SD for at least 6 months [23]. Patients with hemoglobin less than 120 g/L in females and 130 g/L in males were diagnosed with anemia. The neutrophil-to-lymphocyte ratio (NLR) was calculated as the ratio of neutrophil count to lymphocyte count, and patients were categorized into two groups with an NLR cutoff of 3 [24].

### 2.3. Statistical Analysis

The differences among continuous variables were tested using the Mann–Whitney U test, and categorical variables were compared through Pearson’s chi-square test or Fisher’s exact test, depending on the circumstances. PFS and OS were presented using the Kaplan–Meier method with log-rank analysis. Cox regression analysis was performed to calculate the hazard ratio (HR) along with the corresponding 95% confidence interval (CI). In addition, Cox regression analysis was applied to deal with the interference of co-variables on outcomes. Factors with *p* values < 0.1 in univariate Cox regression analysis were added to multivariate analysis. A swimmer plot was applied to present the efficacy and duration of anti-PD1/PD-L1 monotherapy and the subsequent therapy. All analyses were bi-lateral, and the *p* values of less than 0.05 were recognized as statistically significant unless otherwise specified. R software (version 4.0.3) was used to perform all data analyses.

## 3. Results

### 3.1. Characteristics of Patients

Among 318 patients diagnosed with MSI/dMMR stage IV gastrointestinal cancers, 187 received initial anti-PD-1/PD-L1 monotherapy. Of these 187 patients, 91 developed disease progression on PD-1/PD-L1 monotherapy at the data cutoff. Twenty-four patients were excluded since they developed disease progression due to drug discontinuation (*n* = 5), died when the disease progressed (*n* = 7), received subsequent drug therapy plus TACE (*n* = 2), or lacked detailed therapy data (*n* = 10). The remaining 67 patients were analyzed in our study, wherein 26 patients received anti-PD1/PD-L1 plus other drug therapy (ICI-plus group), 25 had chemotherapy with or without targeted therapy (chemo-targeted group), and 16 were treated with BSC (BSC group). The clinicopathological characteristics of the three groups are shown in Table 1. Among the 51 patients who had received post-ICI drug therapy (i.e., the patients in the ICI-plus group and the chemo-targeted group), 40 (78.4%) patients were less than 65 years, 30 (58.8%) patients were male, and 44 (86.3%) patients had an ECOG performance status of 0 or 1. About two-thirds (35, 68.6%) of the patients had CRC, and the other one-third had gastric and gastroesophageal junction (GEJ) cancer (10, 19.6%) and other kinds of gastrointestinal cancer (six, 11.8%). The majority of patients (41, 80.4%) had less than three metastatic organs, with 27 (52.9%) patients having peritoneal metastasis, 24 (47.1%) patients having distant lymph node metastasis, 17 (33.3%) patients having hepatic metastasis, and eight (15.7%) patients having pulmonary metastasis. Most patients (37, 72.5%) had received two or more prior lines of systemic therapy before receiving post-ICI therapy, and approximately half (26, 51.0%) of the patients received post-ICI therapy within one year after disease progression. As for the efficacy of previous anti-PD1/PD-L1 monotherapy, only six (11.8%) patients achieved PR, and 35 (68.6%) patients had primary resistance. Before receiving post-ICI therapy, 25 (49.0%) patients were diagnosed with anemia, and 19 (37.3%) had an NLR of less than 3. The baseline characteristics and clinical benefit from prior anti-PD1/PD-L1 monotherapy were well balanced between the ICI-plus group and chemo-targeted group, except that patients in the chemo-targeted group had received significantly more prior lines of therapy (*p* = 0.006). Compared with the other two groups, the BSC group tended to have more patients with age ≥ 65, female, ECOG ≥ 2, gastric and GEJ cancer, distant lymph node metastasis, and primary resistance to prior anti-PD1/PD-L1 monotherapy (Table 1).

### 3.2. Efficacy

**Figure 2:**
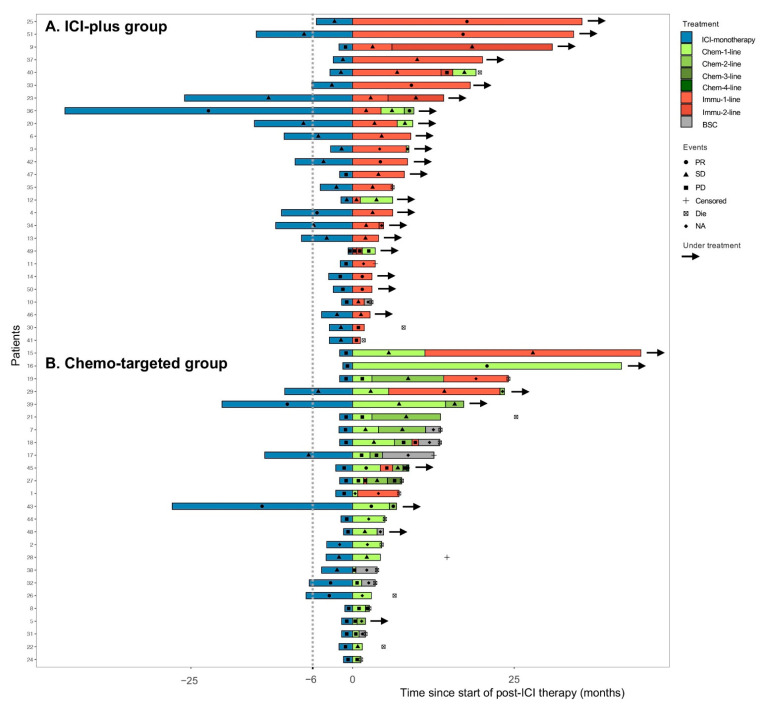
Best response and duration of each line of therapy since the application of anti-PD1/PD-L1 monotherapy in the ICI-plus group (**A**) and chemo-targeted group (**B**). For patients who lacked detailed information about treatment before the date of death or censoring, the graph bar was presented by blank. Abbreviations: ICI-plus: anti-PD1/PD-L1 plus other therapy; chemo-targeted: chemotherapy with or without targeted therapy; BSC: best supportive care; NA = not available; PD = progressive disease; PR = partial response; SD = stable disease.

**Figure 3:**
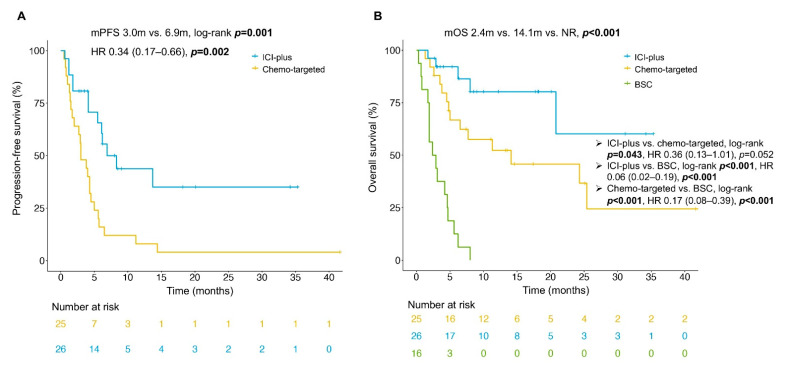
Kaplan–Meier curves of PFS (**A**) and OS (**B**) for MSI/dMMR gastrointestinal cancer patients who received anti-PD1/PD-L1 plus other therapy, chemotherapy with or without targeted therapy and best supporting care (only OS) after progression on prior anti-PD1/PD-L1, respectively. Tick marks mean censored data. The ICI-plus group showed significantly longer PFS (*p* = 0.001) and OS (*p* = 0.043) than the chemo-targeted group with log-rank analysis. The OS of the BSC group was significantly shorter than the other two groups. Abbreviations: ICI-plus: anti-PD1/PD-L1 plus other therapy; chemo-targeted: chemotherapy with or without targeted therapy; BSC: best supportive care; HR = hazard ratio; mOS: median overall survival; mPFS: median progression-free survival; NR = not reached.

For patients who had received post-ICI drug therapy, the median follow-up time was 13.5 months (range, 6.9–20.1 months), and the clinical courses were displayed in Figure 2. The ORR was 23.1% versus 12.0% (*p* = 0.503) and the DCR was 80.8% versus 44.0% (*p* = 0.007) in the ICI-plus group and chemo-targeted group, respectively (Table 2). Compared with the chemo-targeted group, the ICI-plus group achieved significantly better PFS (median PFS [mPFS] 6.9 vs. 3.0 months, log-rank *p* = 0.001), OS (median OS [mOS] not reached [NR] vs.14.1 months, log-rank *p* = 0.043) (Figure 3), and PFSr (2.4 vs. 0.9, *p* = 0.021). The mOS was only 2.4 months for the BSC group, significantly shorter than that for the ICI-plus group (*p* < 0.001) and chemo-targeted group (*p* < 0.001). The 12-month PFS rate and 12-month OS rate in the ICI-plus group were 43.7% and 80.5%, respectively, while the 12-month PFS rate and 12-month OS rate were merely 8.0% and 52.6% in the chemo-targeted group, respectively. It should be pointed out that there were seven patients in the chemo-targeted group who further received ICI-plus therapy after progression, which might have an impact on the comparison of OS between the two groups. After transferring these seven patients from the chemo-targeted group into the ICI-plus group, the benefit of OS in the ICI-plus group over the chemo-targeted-therapy turned more significant (mOS NR vs. 6.5 months, log-rank *p* = 0.008) (Supplement Figure S1).

We further evaluated the favorable prognostic value of ICI-plus therapy by univariate and multivariate Cox regression. As demonstrated in Supplement Table S1, patients who received anti-PD1/PD-L1 plus other therapy had nearly significantly better PFS (HR 0.50, 95% CI 0.24–1.04, *p* = 0.064) and numerically better OS (HR 0.78, 95% CI 0.25–2.41, *p* = 0.664) than patients who received chemotherapy with or without targeted therapy. We found that patients with an ECOG performance status of ≥2 had significantly worse PFS (HR 2.64, 95% CI 0.90–7.71, *p* = 0.077) and numerically worse OS (HR 1.88, 95% CI 0.50–7.09, *p* = 0.350). Moreover, patients with acquired resistance to prior anti-PD1/PD-L1 monotherapy and peritoneal metastasis were related to a better PFS and OS, while female patients seemed to have a worse PFS, and patients with NLR of ≥3 seemed to have a worse OS, though none of the *p* values was significant.

We were interested in the effects of prior anti-PD1/PD-L1 monotherapy on post-ICI drug therapy, and then quantified these effects in terms of PFS, OS, ORR, and DCR. As shown in Supplement Figure S2, compared with the patients who had acquired resistance to prior anti-PD1/PD-L1 monotherapy, patients with primary resistance had a significantly poor OS (mOS, 14.1 months vs. NR, *p* = 0.028) along with a numerically low ORR (14.3% vs. 25%, *p* = 0.592), DCR (54.3% vs. 81.3%, *p* = 0.125), and PFS (mPFS 4.0 months vs. 5.7 months, log-rank *p* = 0.090) from subsequent therapy. These findings also applied to the ICI-plus group. In the ICI-plus group, the ORR (18.8% vs. 30%, *p* = 0.644), DCR (68.8% vs. 100%, *p* = 0.121) were numerically lower in 16 patients with primary resistance than in 10 patients having acquired resistance.

## 4. Discussion

To our knowledge, this was the first retrospective cohort study to compare the efficacy of different treatment strategies after the progression on anti-PD1/PD-L1 monotherapy in MSI/dMMR gastrointestinal cancer patients. The results demonstrated that the ICI-plus group had a significantly better DCR (80.8% vs. 44.0%, *p* = 0.007), PFS (mPFS 6.9 months vs. 3.0 months, log-rank *p* = 0.001), OS (mOS NR vs. 14.1 months, log-rank *p* = 0.043), and PFSr (2.4 vs. 0.9, *p* = 0.021) than the chemo-targeted group, and the BSC group had the worst OS (mOS 2.4 months) among these three groups.

To date, analysis of the suitable therapy options in MSI/dMMR cancer patients after progression on anti-PD1/PD-L1 monotherapy is limited. Generally speaking, there are two kinds of therapy strategies, namely conventional chemotherapy with or without targeted therapy and rechallenge of anti-PD1/PD-L1 with the aid of other therapy. In a retrospective study investigating the efficacy of chemotherapy with or without targeted therapy in MSI/dMMR mCRC patients after progression on anti-PD1/PD-L1 monotherapy [18], the ORR and DCR were 13% and 45%, and the mPFS and mOS were 2.9 months and 7.4 months, respectively, which were consistent with the results in our study. The mOS was 6.5 months in the chemo-targeted group in our study when excluding patients who received anti-PD1/PD-L1 plus other therapy after progression on chemotherapy with or without targeted therapy.

As for continuing anti-PD1/PD-L1 with the combination of other systemic treatments, clinical trials with published findings to date included immunotherapy combined with anti-TGF-β [16] and anti-TIM3 inhibitors [17], respectively. Unfortunately, neither of the results was satisfying. The patients received dual inhibitors of TGF-β and PD-L1 (*n* = 15), yielding an ORR of 0%, DCR of 21%, mPFS of 1.8 months, and mOS of 9.1 months, while the patients received anti-PD-L1 plus anti–TIM-3 antibodies with an ORR of 4.5%, DCR of 31.8%, mPFS of 1.9 months, and mOS of 9.1 months. Remarkably, the efficacy of the ICI-plus group was excellent in our study. According to the results of the KEYNOTE177 trial, the DCR of Pembrolizumab as first-line therapy in MSI/dMMR mCRC patients was 64.7% [7]. However, a slightly higher DCR of 80.8% was achieved in patients receiving anti-PD1/PD-L1 plus other therapy in our study, even though they had progressed on prior anti-PD1/PD-L1 monotherapy. Furthermore, anti-PD1/PD-L1 plus other therapy achieved better ORR (23.1% vs. 7.7%), DCR (80.8% vs. 73.1%), and mPFS (6.9 months vs. 3.7 months) compared with the prior anti-PD1/PD-L1 monotherapy, with a PFSr of 2.4, which was far more than 1.3, on behalf of the benefit of post-ICI therapy compared to prior anti-PD1/PD-L1 monotherapy.

In the ICI-plus group, most patients received ICIs plus anti-angiogenesis therapy (*n* = 15) or chemotherapy (*n* = 9). Preclinical studies have proven that, in CRC patients, anti-angiogenesis therapy could enhance the efficacy of immunotherapy and restrain tumor growth through increasing effector T cell infiltration and decreasing suppressive components within the tumor microenvironment (TME) such as Tregs, myeloid-derived suppressor cells (MDSCs), and M2-tumor-associated macrophages (M2-TAMs) [25,26]. It was surprising to note that through the combination of ICIs and anti-angiogenesis agents, the REGONIVO trial achieved an ORR of 33% in advanced MSS CRC patients [27], whose TME had been thought to be “cold” (lake of immune cells infiltration) and insensitive to ICIs, suggesting the effect of anti-angiogenesis agents on converting TME from immune-suppressive status to immune-supportive status [28]. According to previously published studies, MSI-H CRC patients generally had higher serum concentrations of vascular endothelial growth factor (VEGF) compared with MSS CRC patients [29,30], indicating that ICIs plus anti-angiogenesis agents might be promising in MSI/dMMR CRC patients. Moreover, it was also found in a prior study that MSI/dMMR gastrointestinal cancer patients who had a poor response and clinical outcomes to ICI therapy tended to have upregulations in the pathways of angiogenesis with a high expression of VEGF-A [31]. The ongoing COMMIT trial (NCT02997228) is evaluating the efficacy of atezolizumab, alone or in combination with chemotherapy, plus bevacizumab as first-line therapy in MSI/dMMR CRC, and we look forward a lot to its results.

There were only two patients receiving anti-PD1/PD-L1 plus anti-CTLA4 antibodies in our study, one had the best response of SD and progressed after four months of therapy, the other one achieved PR after therapy and had a durable response over thirty-four months at data cutoff. Actually, the combination strategy of anti-PD1/PD-L1 and anti-CTLA4 antibodies was promising. There were several case reports demonstrating the efficacy of anti-PD1/PD-L1 plus anti-CTLA4 antibodies after ICI progression [32,33,34,35,36]. Even though most patients were heavily pretreated, altogether four patients of seven patients achieved the best response of PR, among whom one patient achieved a durable response for as long as almost two years. These results demonstrated the promising value of anti-PD1/PD-L1 plus anti-CTLA4 antibodies, although the bias of publication should be considered. Nevertheless, considering the serious immune-related adverse events reported in prior studies [37], caution must be taken when applying anti-PD1/PD-L1 plus anti-CTLA4 antibodies, especially in those who had immune-related adverse events in prior anti-PD1/PD-L1 monotherapy.

In the multivariate Cox analysis, a significantly better PFS was observed in patients receiving anti-PD1/PD-L1 plus other therapy versus conventional chemotherapy with or without targeted therapy (HR 0.50, 95% CI 0.24–1.04, *p* = 0.064). The advantage of anti-PD1/PD-L1 plus other therapy versus conventional chemotherapy with or without targeted therapy of improving OS was also observed, though the *p* value was not significant, which might partly result from the small sample size and the cross of therapy (HR 0.78, 95% CI 0.25–2.41, *p* = 0.664). Consistent with the discovery of prior studies [38], we also found that a worse ECOG performance seemed to negatively impact PFS and OS. Patients with acquired resistance to prior anti-PD1/PD-L1 monotherapy were related to better PFS and OS, while the best response to prior anti-PD1/PD-L1 seemingly had no impact on PFS or OS. Unexpectedly, peritoneal metastasis seemed to have a positive effect on PFS and OS, contradicting common sense. We tended to ascribe the results to the small sample size-driven instability of the analysis model. Actually, it was reported that patients with peritoneal metastases and ascites had worse PFS and OS than those without peritoneal metastases and those with peritoneal metastases and no ascites when receiving ICI-based therapy [39].

There are several limitations to our study. Firstly, it was a retrospective study, and the follow-up time was not long enough to produce sufficient events. Multiple gastrointestinal cancer types and combination immunotherapy strategies were included in this study. However, analysis according to cancer types and therapy strategies was hard to perform in our study due to the sample size restriction. Moreover, information on adverse events was not collected in our study, but the safety of combination immunotherapy was proven in many previous studies. Lastly, the study population was limited to patients who progressed on anti-PD1/PD-L1 monotherapy; therefore, its findings may not be generalizable to other patient populations, such as those who progressed on anti-PD1/PD-L1 plus anti-CTLA4 antibodies.

## 5. Conclusions

The key contribution of this work is that we first compare the efficacy of different post-ICI therapy strategies in MSI/dMMR gastrointestinal cancer patients with a relatively large sample size from multiple centers. After the progression of anti-PD1/PD-L1 monotherapy, the combination of anti-PD1/PD-L1 with other therapy presented significantly better efficacy than conventional chemotherapy with or without targeted therapy in MSI/dMMR gastrointestinal cancer patients, which should be validated in further clinical trials.

## Figures and Tables

**Table 1:** Baseline characteristics of patients.

Characteristics	All Patients with Post-ICI Therapy *n* = 51	ICI-Plus Group *n* = 26	Chemo-Targeted Group *n* = 25	*p* Value	BSC Group *n* = 16
Age					
<65	40 (78.4)	21 (80.8)	19 (76.0)	0.941	8 (50.0)
≥65	11 (21.6)	5 (19.2)	6 (24.0)		8 (50.0)
Sex					
Female	21 (41.2)	8 (30.8)	13 (52.0)	0.124	10 (62.5)
Male	30 (58.8)	18 (69.2)	12 (48.0)		6 (37.5)
ECOG					
0–1	44 (86.3)	22 (84.6)	22 (88.0)	0.962	8 (50.0)
≥2	5 (9.8)	2 (7.7)	3 (12.0)		8 (50.0)
NA	2 (3.9)	2 (7.7)	0 (0.0)		0 (0.0)
Differentiation					
Moderate-high	19 (37.3)	11 (42.3)	8 (32.0)	0.376	5 (31.3)
Undifferentiation-low	29 (56.9)	13 (50.0)	16 (64.0)		10 (62.5)
NA	3 (5.9)	2 (7.7)	1 (4.0)		1 (6.3)
Site					
Gastric and GEJ	10 (19.6)	4 (15.4)	6 (24.0)	0.241	7 (43.8)
Colorectal	35 (68.6)	17 (65.4)	18 (72.0)		7 (43.8)
Other sites	6 (11.8)	5 (19.2)	1 (4.0)		2 (12.5)
No. of metastatic organs					
<3	41 (80.4)	22 (84.6)	19 (76.0)	0.673	11 (68.8)
≥3	10 (19.6)	4 (15.4)	6 (24.0)		5 (31.3)
Hepatic metastasis					
Absent	34 (66.7)	18 (69.2)	16 (64.0)	0.692	10 (62.5)
Present	17 (33.3)	8 (30.8)	9 (36.0)		6 (37.5)
Pulmonary metastasis					
Absent	43 (84.3)	24 (92.3)	19 (76.0)	0.224	14 (87.5)
Present	8 (15.7)	2 (7.7)	6 (24.0)		2 (12.5)
Distant lymph node metastasis					
Absent	27 (52.9)	14 (53.8)	13 (52.0)	0.895	5 (31.3)
Present	24 (47.1)	12 (46.2)	12 (48.0)		11 (68.8)
Peritoneal metastasis					
Absent	24 (47.1)	11 (42.3)	13 (52.0)	0.488	7 (43.8)
Present	27 (52.9)	15 (57.7)	12 (48.0)		9 (56.3)
No. of prior therapy lines					
<2	14 (27.5)	12 (46.2)	2 (8.0)	0.006	5 (31.3)
≥2	37 (72.5)	14 (53.8)	23 (92.0)		11 (68.8)
Time from metastasis to post-ICI therapy					
<12 months	26 (51.0)	12 (46.2)	13 (52.0)	0.886	-
≥12 months	25 (49.0)	14 (53.8)	12 (48.0)		-
Best response to prior anti-PD1/PD-L1					
PR	6 (11.8)	2 (7.7)	4 (16.0)	0.590	1 (6.3)
SD + PD	44 (86.3)	24 (92.3)	20 (80.0)		15 (93.8)
NA	1 (2.0)	0 (0.0)	1 (4.0)		0 (0.0)
Type of resistance					
primary resistance	35 (68.6)	16 (61.5)	19 (76.0)	0.406	14 (87.5)
acquired resistance	16 (31.4)	10 (38.5)	6 (24.0)		2 (12.5)
Anemia					
Absent	18 (35.3)	10 (38.5)	8 (32.0)	0.455	-
Present	25 (49.0)	11 (42.3)	14 (56.0)		
NA	8 (15.7)	5 (19.2)	3 (12.0)		
NLR					
<3	19 (37.3)	9 (34.6)	10 (40.0)	0.864	-
≥3	24 (47.1)	12 (46.2)	12 (48.0)		
NA	8 (15.7)	5 (19.2)	3 (12.0)		

Data are *n* (%). Abbreviations: ICI-plus: anti-PD1/PD-L1 plus other therapy; chemo-targeted: chemotherapy with or without targeted therapy; BSC: best supportive care; ECOG = Eastern Cooperative Oncology Group; GEJ = gastroesophageal junction; ICI = immune checkpoint inhibitor; NA = not available; NLR = neutrophil-to-lymphocyte ratio; No. = number; PD = progressive disease; PD1 = programmed death 1; PD-L1 = programmed death ligand-1; PR = partial response; SD = stable disease.

**Table 2:** Overall response between the ICI-plus group and chemo-targeted group.

Variable	ICI-Plus Group *n* = 26	Chemo-Targeted Group *n* = 25	*p* Value
Best response, *n* (%)			
Complete response	0 (0.0)	0 (0.0)	
Partial response	6 (23.1)	3 (12.0)	
Stable disease	15 (57.7)	8 (32.0)	
Progressive disease	3 (11.5)	10 (40.0)	
Unable to determine *	2 (7.7)	4 (16.0)	
Objective response, *n* (%)	6 (23.1)	3 (12.0)	0.503
Disease control, *n* (%)	21 (80.8)	11 (44.0)	0.007

* These patients had no post-baseline efficacy evaluation due to various reasons. Data are *n* (%). Abbreviations: ICI-plus: anti-PD1/PD-L1 plus other therapy; chemo-targeted: chemotherapy with or without targeted therapy.

## Data Availability

Data are available upon reasonable request.

## Supplementary Materials

The following supporting information can be downloaded at: https://www.mdpi.com/article/10.3390/cancers14205158/s1, Figure S1: Kaplan–Meier curves of OS for MSI/dMMR gastrointestinal cancer patients who received anti-PD1/PD-L1 plus other therapy (no matter in which line) and chemotherapy with or without targeted therapy after progression on prior anti-PD1/PD-L1, respectively; Figure S2: Kaplan–Meier curves of PFS (A) and OS (B) for MSI/dMMR gastrointestinal cancer patients who had primary and acquired resistance to anti-PD1/PD-L1 monotherapy, respectively; Table S1: Univariate and multivariate Cox regression analysis of factors related to PFS and OS.
