# Supplementary material for: The Optimal Therapy after Progression on Immune Checkpoint Inhibitors in MSI Metastatic Gastrointestinal Cancer Patients: A Multicenter Retrospective Cohort Study
Source: Cancers (Basel). 2022 Oct 21;14(20):5158. doi: 10.3390/cancers14205158 (PMC9601260; doi:10.3390/cancers14205158)
Supplement: Supplementary file 1 [file cancers-14-05158-s001.zip › cancers-1937864-supplementary.pdf]

Online-Only Supplement

Supplement to: Chen, et al. The Optimal Therapy after Progression on Immune Checkpoint Inhibitors in MSI Metastatic Gastrointestinal Cancer Patients: A Multicenter Retrospective Cohort Study

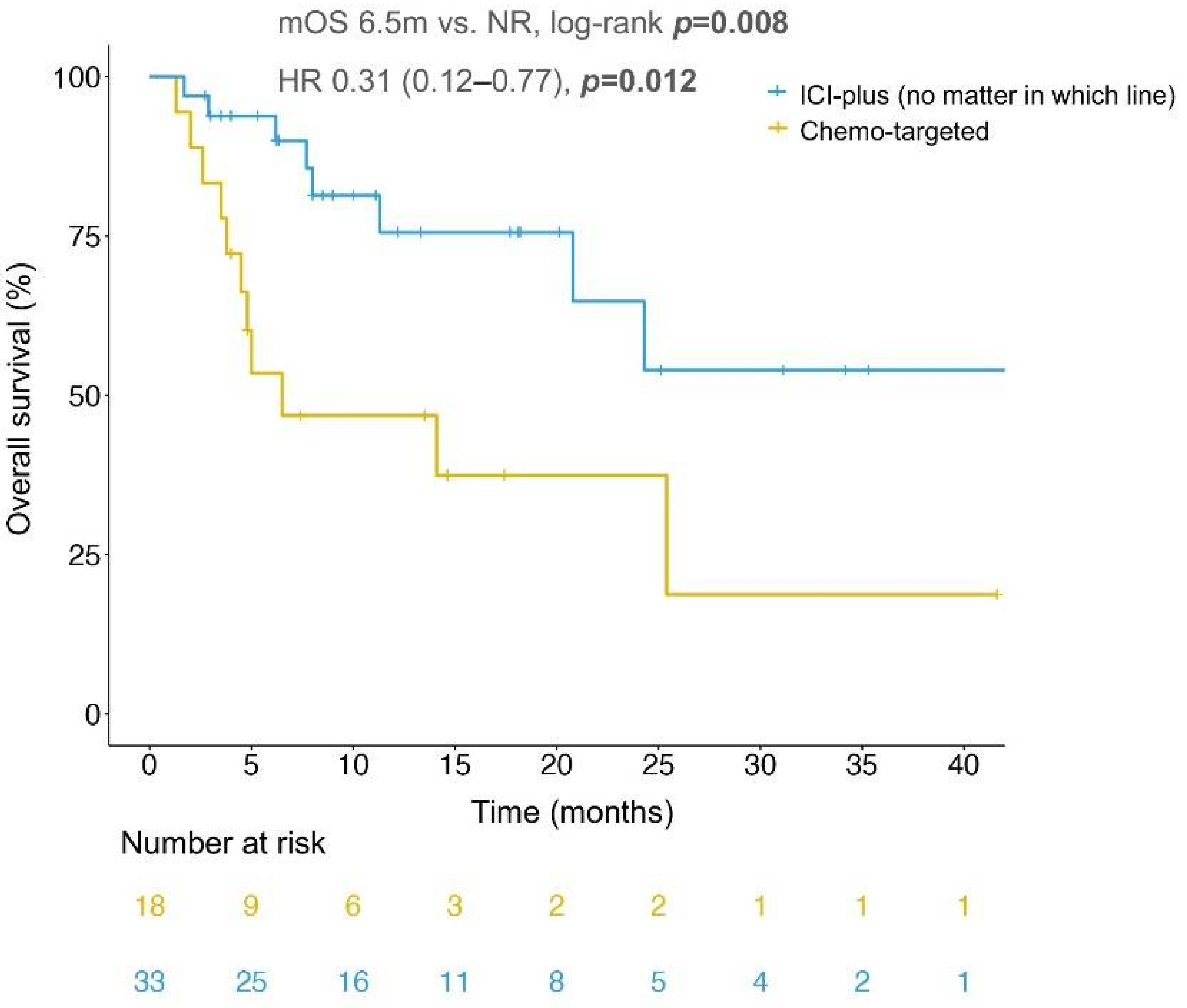

Supplement Figure S1. Kaplan–Meier curves of OS for MSI/dMMR gastrointestinal cancer patients who received anti-PD1/PD-L1 plus other therapy (no matter in which line) and chemotherapy with or without targeted therapy after progression on prior anti-PD1/PD-L1, respectively. Tick marks mean censored data. Patients who received anti-PD1/PD-L1 plus other therapy (no matter in which line) showed significantly better OS (*P*=0.008) than patients who received chemotherapy with or without targeted therapy. Abbreviations: HR: hazard ratio; mOS: median overall survival; NR: not reached; ICI-plus: anti-PD1/PD-L1 plus other therapy; chemo-targeted: chemotherapy with or without targeted therapy.

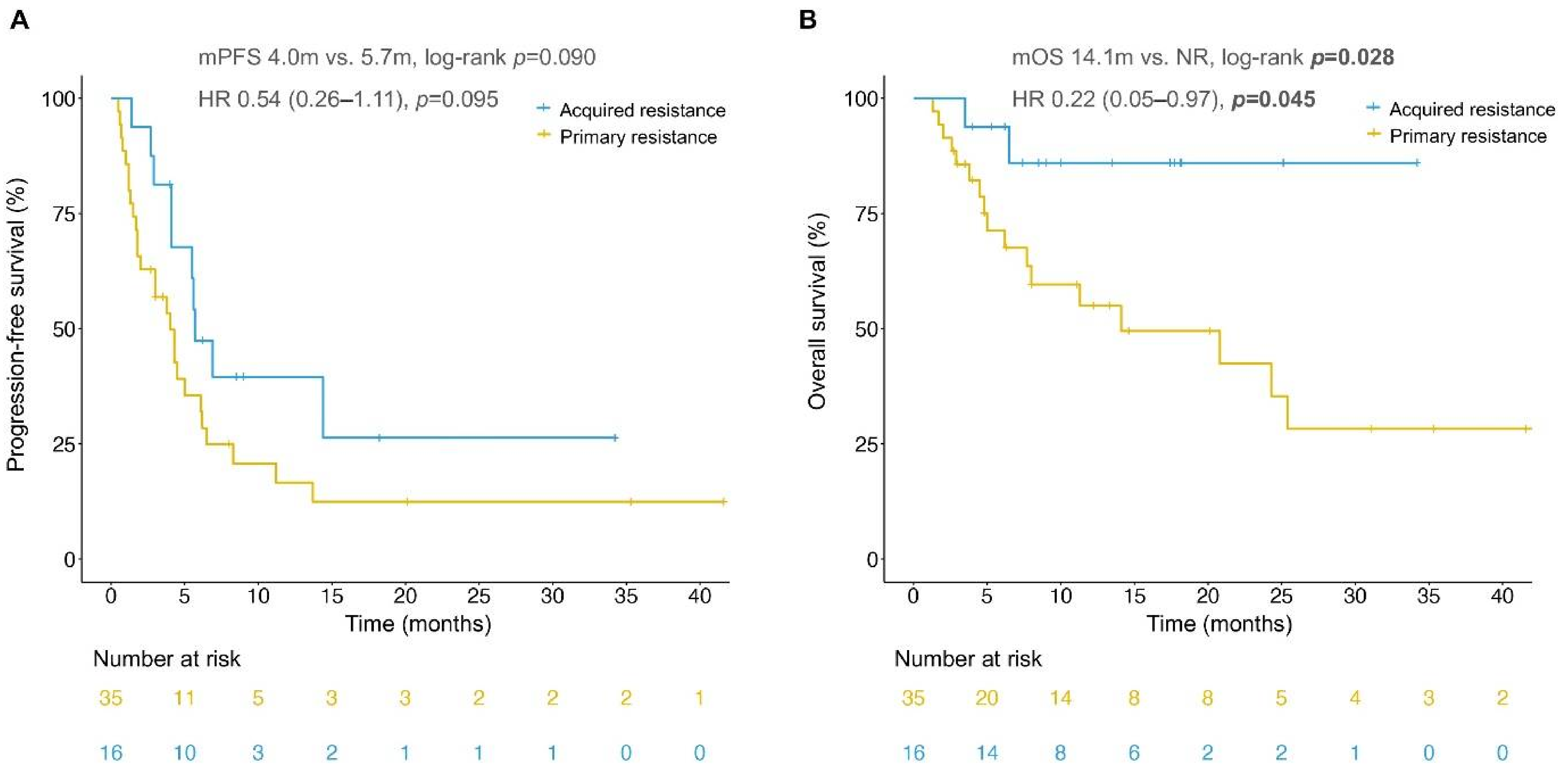

Supplement Figure S2. Kaplan–Meier curves of PFS (A) and OS (B) for MSI/dMMR gastrointestinal cancer patients with primary and acquired resistance to anti-PD1/PD-L1 monotherapy, respectively. Tick marks mean censored data. Patients who had acquired resistance showed numerically longer PFS (*P*=0.090) and significantly longer OS (*P*=0.028) than patients who had primary resistance. Abbreviations: HR: hazard ratio; NR: not reached; mPFS: median progression-free survival; mOS: median overall survival.

Supplement Table S1. Univariate and multivariate Cox regression analysis of factors related to PFS and OS.

Parameter	PFS				OS			
	Univariate analysis		Multivariate analysis		Univariate analysis		Multivariate analysis	
	HR (95% CI)	*P* value	HR (95% CI)	*P* value	HR (95% CI)	*P* value	HR (95% CI)	*P* value
Anti-PD1/PD-L1 plus other therapy	0.34 (0.17–0.66)	**0.002**	0.50 (0.24–1.04)	0.064	0.36 (0.13–1.01)	0.052	0.78 (0.25–2.41)	0.664
Age ≥ 65	1.57 (0.74–3.34)	0.240			1.68 (0.54–5.23)	0.369		
Female sex	1.83 (0.95–3.51)	0.071	1.40 (0.68–2.89)	0.360	1.89 (0.77–4.67)	0.167		
ECOG ≥ 2	4.60 (1.64–12.90)	**0.004**	2.64 (0.90–7.71)	0.077	5.59 (1.74–17.93)	**0.004**	1.88 (0.50–7.09)	0.350
Undifferentiation-low differentiation	1.18 (0.60–2.33)	0.639			0.68 (0.26–1.78)	0.432		
Site								
Gastric and GEJ	Ref.				Ref.			
Colorectal	0.87 (0.39–1.94)	1.000			0.94 (0.27–3.34)	0.762		
Other sites	0.69 (0.21–2.31)	0.620			0.49 (0.05–4.71)	0.509		
≥ 3 metastatic sites	1.58 (0.71–3.55)	0.264			1.36 (0.44–4.16)	0.591		
Hepatic metastasis	1.28 (0.64–2.57)	0.480			1.87 (0.69–5.08)	0.218		
Pulmonary metastasis	1.50 (0.64–3.51)	0.346			0.85 (0.25–2.92)	0.795		
Lymph node metastasis	1.72 (0.88–3.34)	0.110			1.06 (0.43–2.62)	0.895		
Peritoneal metastasis	0.53 (0.27–1.03)	0.061	0.57 (0.28–1.16)	0.124	0.33 (0.12–0.89)	**0.029**	0.51 (0.16–1.63)	0.259
≥ 2 prior treatment lines	0.93 (0.44–1.98)	0.852			1.26 (0.36–4.41)	0.713		
Time from metastasis to post-ICI therapy > 12 months	0.94 (0.49–1.80)	0.843			0.70 (0.28–1.75)	0.447		
PR as the best response to prior anti-PD1/PD-L1	1.06 (0.41–2.74)	0.902			1.17 (0.26–5.24)	0.835		
Acquired resistance to prior anti-PD1/PD-L1	0.54 (0.26–1.11)	0.095	0.61 (0.29–1.30)	0.200	0.22 (0.05–0.97)	**0.045**	0.23 (0.03–1.91)	0.174
Anemia	1.18 (0.58–2.42)	0.647			2.08 (0.75–5.76)	0.159		
NLR ≥ 3	1.36 (0.67–2.77)	0.395			6.77 (1.54–29.72)	**0.011**	3.73 (0.78–17.90)	0.101

Abbreviations: CI: confidence interval; ECOG: Eastern Cooperative Oncology Group; GEJ: gastroesophageal junction; HR: hazard ratio; ICI: immune checkpoint inhibitor; NLR: neutrophil to lymphocyte ratio; OS: overall survival; PD1: programmed death 1; PD-L1: programmed death ligand-1; PFS: progression-free survival; PR: partial response.
